# Using Atrial Fibrillation Burden Trends and Machine Learning to Predict Near-Term Risk of Cardiovascular Hospitalization

**DOI:** 10.1161/CIRCEP.124.012991

**Published:** 2024-10-24

**Authors:** James Peacock, Evan J. Stanelle, Lawrence C. Johnson, Elaine M. Hylek, Rahul Kanwar, Dhanunjaya R. Lakkireddy, Suneet Mittal, Rod S. Passman, Andrea M. Russo, Dana Soderlund, Mellanie True Hills, Jonathan P. Piccini

**Affiliations:** White Plains Hospital, NY (J.P.).; Medtronic, Inc, Minneapolis, MN (E.J.S., L.C.J., R.K., D.S.).; Boston University Medical Center, MA (E.M.H.).; The Kansas City Heart Rhythm Institute and Research Foundation, Overland Park (D.R.L.).; Valley Health System, Ridgewood, NJ (S.M.).; Division of Cardiology, Northwestern University, Feinberg School of Medicine, Chicago, IL (R.S.P.).; Electrophysiology and Arrhythmia Services, Cooper University Hospital, Camden, NJ (A.M.R.).; StopAfib.org, American Foundation for Women’s Health, Decatur, TX (M.T.H.).; Duke Clinical Research Institute and Duke University Medical Center, Durham, NC (J.P.P.).

**Keywords:** atrial fibrillation, data warehousing, hospitals, humans, risk

## Abstract

**BACKGROUND:**

Atrial fibrillation is associated with an increased risk of cardiovascular hospitalization (CVH), which may be triggered by changes in daily burden. Machine learning of dynamic trends in atrial fibrillation burden, as measured by insertable cardiac monitors (ICMs), may be useful in predicting near-term CVH.

**METHODS:**

Using Optum’s deidentified Clinformatics Data Mart Database (2007–2019), linked with the Medtronic CareLink ICM database, we identified patients with >1 days of ICM-detected atrial fibrillation. ICM-detected diagnostic parameters were transformed into simple moving averages over different periods for daily follow-up. A diagnostic trend was defined as the comparison of 2 simple moving averages of different periods for each diagnostic parameter. CVH was defined as any hospital, emergency department, or ambulatory surgical center encounter with a cardiovascular diagnosis-related group or diagnosis code. Machine learning was used to determine which diagnostic trends could best predict patient risk 5 days before CVH.

**RESULTS:**

A total of 2616 patients with ICMs met the inclusion criteria (71±11 years; 55% male). Among them, 1998 (76%) had a planned or unplanned CVH over 605 363 days. Machine learning revealed distinct groups: (A) sinus rhythm (reference), (B) below-average burden, (C) above-average burden, and (D) above-average burden with decreasing patient activity. The relative risk was increased in all groups versus the reference (B, 4.49 [95% CI, 3.74–5.40]; C, 8.41 [95% CI, 7.00–10.11]; D, 11.15 [95% CI, 9.10–13.65]), including a 21% increase in CVH detection over prespecified burden thresholds of duration (≥1 hour) and quantity (≥5%). The area under the receiver operating characteristic curve increased from 0.55 when using hourly burden amounts to 0.66 when using burden trends and decreasing patient activity (*P*<0.001), a 20% increase in predictive power.

**CONCLUSIONS:**

Trends in atrial fibrillation were strongly associated with near-term CVH, especially above-average burden coupled with low patient activity. This approach could provide actionable information to guide treatment and reduce CVH.

**WHAT IS KNOWN?**

- Long-term cardiac monitoring with continuous monitoring devices has shown that atrial fibrillation (AF) burden, typically defined as the total amount of AF within a defined monitoring period, is positively associated with stroke and cardiovascular hospitalization.
- The clinical progression of AF from shorter to longer duration episodes is associated with increased risks of stroke and death.

**WHAT THE STUDY ADDS**

- Long-term continuous monitoring of AF allows tracking not only the total burden of AF but also the detection of above-average changes in AF burden over time. This may help identify patients at risk for adverse cardiovascular outcomes and hospitalization.
- An increasing trend in AF burden and low levels of insertable cardiac monitor–detected patient activity, when combined with higher levels of burden duration or quantity, result in the highest risk segments for near-term cardiovascular hospitalization.
- Individualized risk prediction, as measured by an increase in insertable cardiac monitor–detected AF burden and low levels of insertable cardiac monitor–detected patient activity, is both an independent predictor of near-term cardiovascular hospitalization and a modifier of the current AF burden risk paradigm.

Historically, atrial fibrillation (AF) has been classified by type (paroxysmal, persistent, or permanent), with accumulating evidence suggesting that patients with persistent or permanent AF are at a higher risk of stroke compared with those with paroxysmal AF. Previous studies have demonstrated that the elevated risk of stroke in patients with persistent or permanent AF may be attributed to the longer durations of AF compared with the shorter, intermittent durations characteristic of paroxysmal AF.^1^ Long-term cardiac monitoring with continuous monitoring devices has shown that AF burden, typically defined as the total amount of AF within a defined monitoring period, is positively associated with stroke^2^ and cardiovascular hospitalization (CVH).^3^ While the temporal relationship between AF incidence and CVH is unknown, the clinical progression of AF from shorter to longer duration episodes is associated with increased risks of stroke and death.^4^ More recently, AF burden progression was shown to occur in patients with cardiac implantable electronic devices within a short time window preceding death.^5^ Leveraging long-term cardiac monitoring solutions, such as insertable cardiac monitors (ICMs), to substitute categorical definitions of AF with dynamic measures of AF burden^1^ may offer additional prognostic value for the personalized risk stratification of AF-related outcomes among patients with AF.

In the present study, we assessed dynamic trends in ICM-detected diagnostic parameters by comparing changes in moving averages and their association with the risk of near-term CVH. Our approach involves a change-from-baseline method, which personalizes the relative risk (RR) of CVH based on changes in diagnostic trends for each patient. This allows for time-updated risk calculations at each daily follow-up through continuous monitoring. Machine learning was utilized to identify which specific diagnostic trends are optimal for predicting the near-term risk of CVH. The objectives of this analysis were to (1) describe an AF burden trend at high risk for near-term CVH and (2) compare its association with near-term CVH with similar measures for AF burden thresholds of duration (≥1 hour) and quantity (≥5%).

## Methods

We conducted a retrospective cohort study using nationwide clinical practice data from Optum’s deidentified Clinformatics Data Mart Database (2007–2019), which contains claims data (commercial and Medicare Advantage health plan data) from multiple hospitals in the United States, linked to the Medtronic CareLink database of ICMs. Data sets were deidentified before analysis, as defined by HIPAA in 45 CFR Section 164.514(b) and were thus exempt from institutional review board review. In accordance with the contractual arrangement between Medtronic, Inc, and Optum, the data cannot be made available to other researchers for the purposes of reproducing the results or replicating the procedure.

### Study Cohort

**Figure 1.**
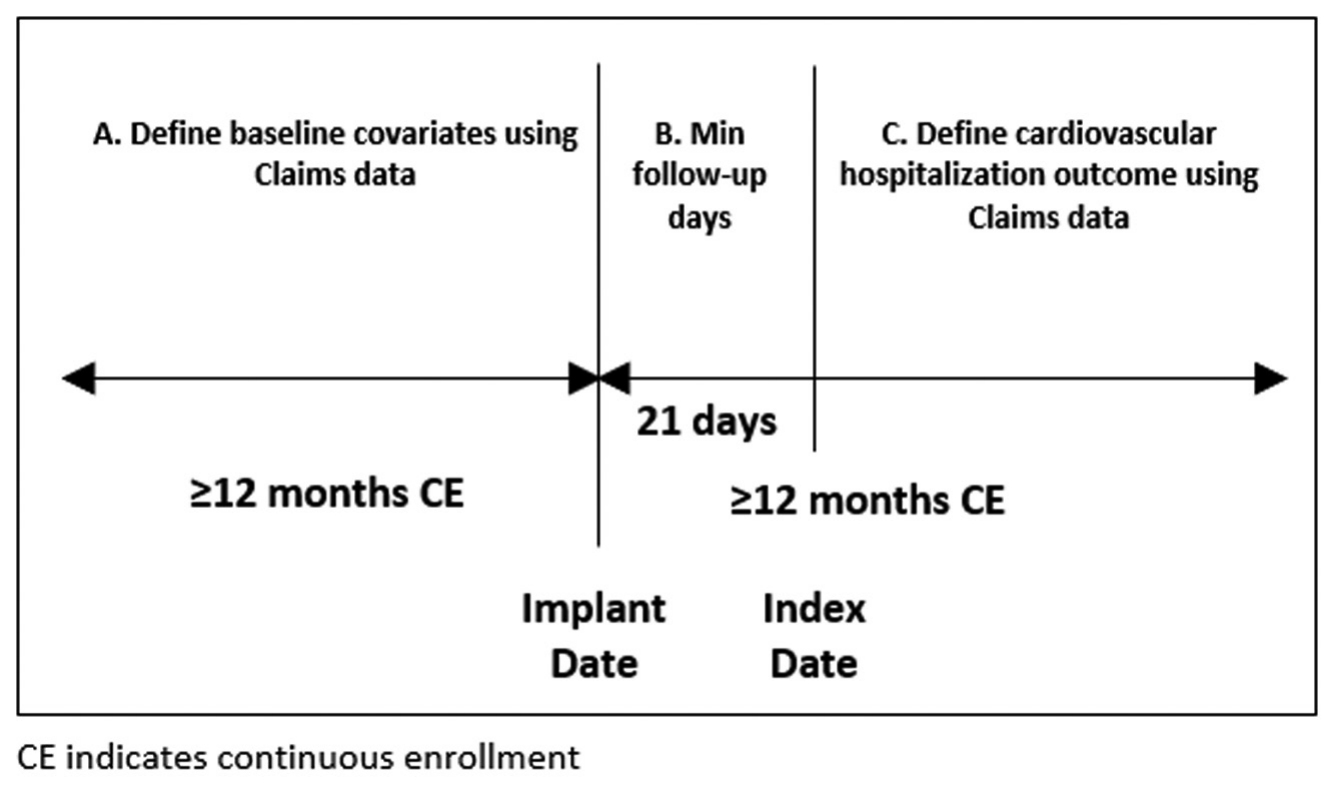
**Definition of the index date.** CE indicates continuous enrollment.

Patients were included if they had a Medtronic Reveal XT or Reveal LINQ ICM with a corresponding device indication for AF management, clinical concern for suspected AF, or the occurrence of cryptogenic stroke as determined by the implanting physician. Patients 18 years and older, without dual Medicare and Medicaid eligibility, were included if they had at least 12 months of continuous claims enrollment between the first day of their respective eligibility start month and the last day of their respective eligibility end month, before and after device implantation. An index date was nominally assigned at 21 days after ICM implantation to initialize a time series for each diagnostic device parameter (Figure 1). Patients were included if they had at least 1 follow-up day of device-detected atrial tachycardia (AT)/AF burden >0 on or after the index date and were excluded if they had a gap in daily follow-up of ≥30 days. Patients with a CVH post-implantation were included if the event occurred during continuous enrollment (Figure 1, period C).

### Derivation of Claims Data

Table S1 presents the diagnosis and procedure codes for each disease. A patient was considered to have a qualifying claims history if there was an acute, inpatient, diagnosis code date or 2 outpatient diagnosis code dates, separated by at least 30 days, associated with the patient before the implant date. CHA_2_DS_2_-VASc scores were calculated according to a patient’s claims history before the ICM implant date using the code list in Table S1. Anticoagulation therapy was defined as a binary variable indicating a claims history of an oral anticoagulation prescription before ICM implantation (Table S2).

### Outcomes Ascertainment

The occurrence of CVH as a study end point was obtained from claims data. A patient was labeled as having an occurrence of planned or unplanned CVH if either of the following was true after the index date:

1. The patient received a diagnosis code (Table S1) or a diagnosis-related group (Table S3) code for CVH (215–316) from an inpatient or outpatient hospital, emergency department, or ambulatory surgical center (place of service equal to 21, 22, 23, or 24, respectively).
2. The patient had an inpatient confinement with a diagnosis code or diagnosis-related group code for CVH.

Only the first occurrence of CVH was recorded if a patient had multiple diagnoses or diagnosis-related group codes.

### Derivation of Diagnostic Data

Diagnostic parameters included daily total AT/AF burden (hours/day), total patient activity (minutes/day), average ventricular rate (beats per minute) measured from midnight to 4:00 am (night) and from 8:00 am to 8:00 pm (day), and heart rate variability (measured as the SD of the 5-minute RR medians over a 24-hour period). The minimum detection threshold for the device to register an AT/AF event was 2 minutes.^6^ Patients were classified into persistent and paroxysmal groups based on device-detected AT/AF amounts collected on or after the index date. Patients with ≥23.5 hours of AT/AF for 7 consecutive days or more were classified as persistent. Patients not classified as persistent and having at least 2 minutes of device-detected AT/AF were classified as paroxysmal. Missing data resulting from a gap in daily follow-up was interpolated by forward-filling the last known value for each diagnostic parameter. Follow-up was limited to 2 years unless there was a CVH, in which case follow-up ended the day before the event.

To determine diagnostic trends, each device parameter was evaluated as a cumulative moving average from the day after implantation and as a simple moving average of different clinical windows (1, 2, 3, 5, 8, 13, and 21 days) starting 21 days after implantation. For device parameter *d* at time *t*:


$$\begin{array}{l} CMA=\frac{1}{t}\underset{i=1}{\overset{t}{\sum }}\;d_{i} \end{array}$$

$$\begin{array}{l} SMA_{p}=\frac{1}{p}\underset{i=t-p+1}{\overset{t}{\sum }}\;d_{i} \end{array}$$

where *t* denotes days after implantation and *p* denotes the simple moving average period. A prior study investigating the short-term effects of daily AF burden on heart failure showed that a short-term trend in AF burden, defined as taking the offset between the 7-period simple moving average and the cumulative moving average, was a reliable risk factor for HF.^7^ Extending this methodology to the present study, the moving average of each clinical window was compared with the other moving averages to create a unique combination of dynamic trends for each device parameter. For example, an 8-day moving average value less than the corresponding 21-day moving average at follow-up would suggest a dynamic, decreasing trend in the given device parameter.

For AF burden, prespecified clinical thresholds were also defined. A duration threshold was defined as any follow-up day with a continuous AF duration ≥1 hour, regardless of when the episode started. A quantity threshold was defined as any follow-up day with a total AT/AF amount ≥72 (5%) minutes. These prespecified thresholds served as clinical comparators for AF burden trends.

### Diagnostic Trend Modeling

Machine learning analysis was performed to identify and describe which diagnostic trends are associated with CVH. A random forest methodology incorporating a bootstrap of 70%/30% learning/test partitions (Supplemental Methods S1 and S2) was applied to the data to determine which device parameter values and trends could reliably classify the 5 days preceding an occurrence of CVH. Patients were then randomly divided into 2 groups: a 70% partition for defining a diagnostic trend and a 30% holdout for validating its performance. To define a high-risk trend, the incidence rate of CVH and the percentage of patients were calculated for each rule in the random forest and for the prespecified duration and quantity thresholds. Diagnostic trends from the random forest output were then matched to these clinical thresholds by incidence rate and patient percentage and applied to the holdout sample for validation. Validation results were calculated using descriptive statistics, RR ratios, area under the receiver operating characteristic curve (AUROC), and tests for equal proportions. *P*<0.05 was considered significant. All analyses were performed using R software, version 4.1.3.

## Results

### Baseline Characteristics

There were 83 357 patients with ICM with linked claims and CareLink data. Of these, 2616 patients met our inclusion criteria (Figure S1), including 2512 (96%) Reveal LINQ devices and 104 (4%) Reveal XT devices. In total, 1998 (76%) patients experienced planned or unplanned CVH post-implant. All patients in our cohort had at least 1 day with device-detected AT/AF, including 160 (6%) patients who experienced persistent AF and 2456 (94%) who experienced paroxysmal AF. The mean daily AF burden was 65±262 minutes.

Table 1 presents the baseline characteristics of the patients according to the occurrence of CVH. Overall, the cohort was 55% male, with a mean age of 71.0±11.0 years. Patients who experienced a CVH had higher rates for all comorbidities except systemic embolism, vascular disease, myocardial infarction, and hyperthyroidism. Patients with CVH following device implantation were more likely to have a history of AF (64% versus 47%; *P*<0.001) and heart failure (20% versus 8%; *P*<0.001) per diagnosis codes recorded before implant.

**Table 1.**
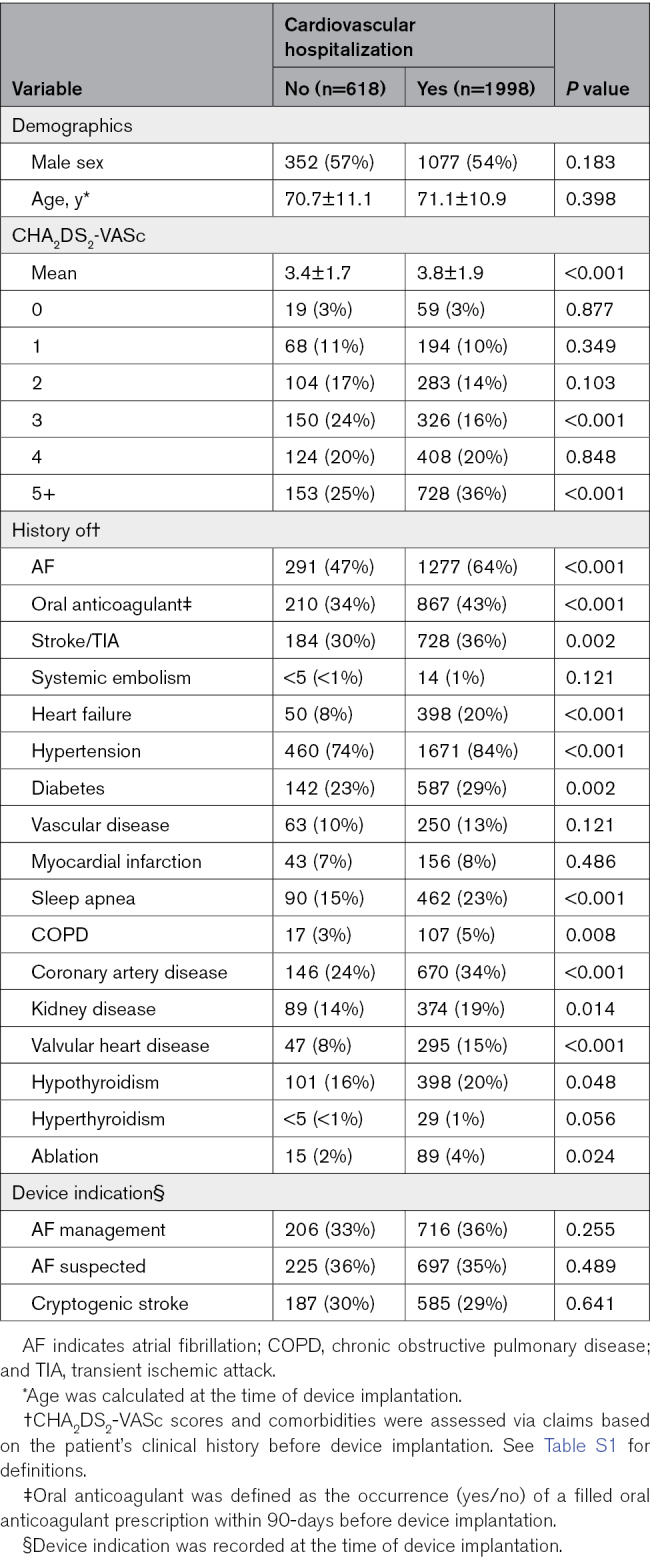
Baseline Demographics

### Follow-Up

There were 605 363 total patient days of follow-up, with an average of 139±156 days for patients with a health care event and 532±213 days for patients without. In total, 233 patients (9%) had a gap in follow-up, with an average gap of 7.4±7 days. Patients with CVH were less likely to have a gap in follow-up (5% versus 23%; *P*<0.001). Of those patients with CVH who experienced a gap, there was no statistical difference in average gap size versus those without CVH (7.42 versus 7.38 days; *P*=0.956).

### AF Burden Trends

**Figure 2.**
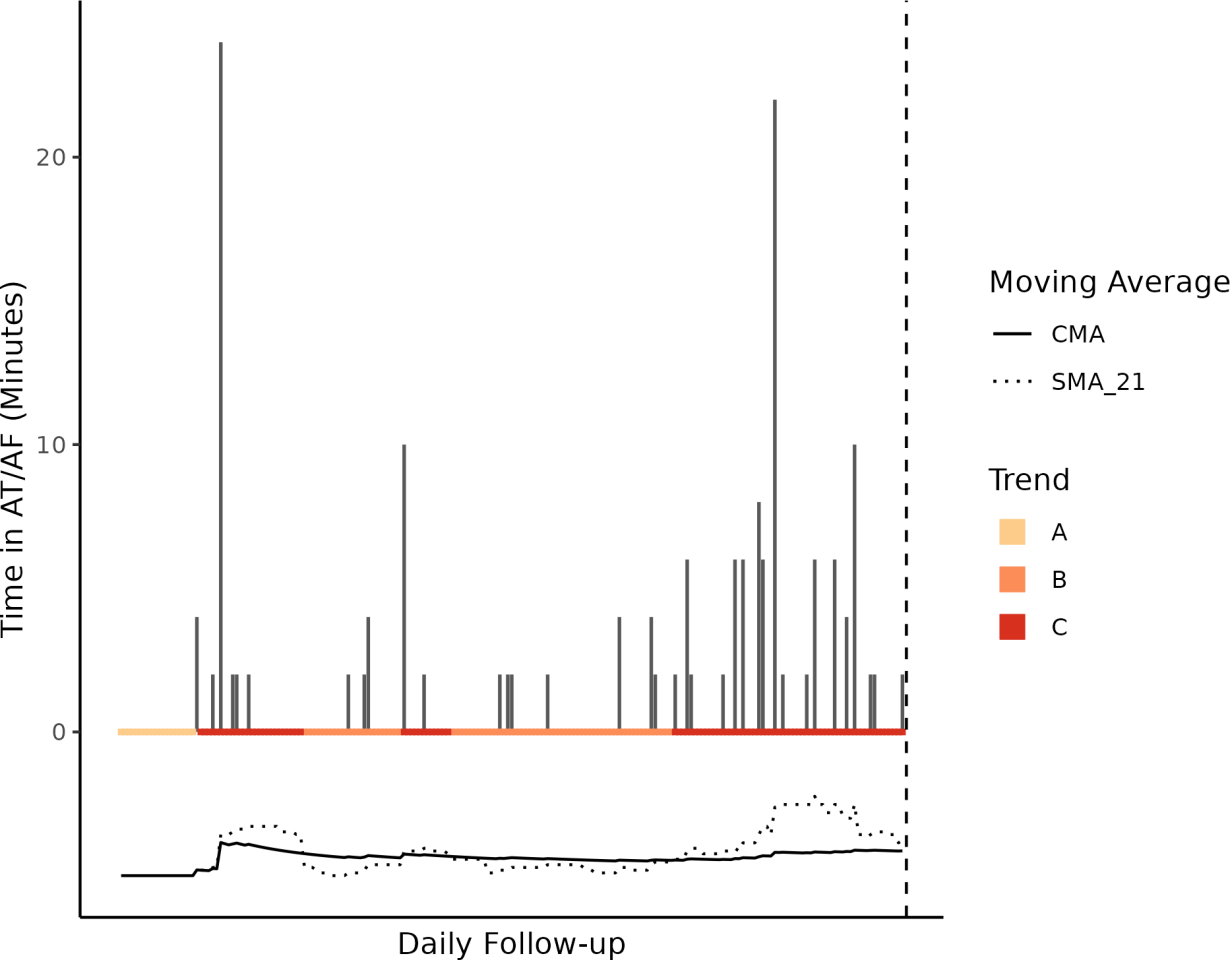
**Mapping atrial fibrillation (AF) burden trends to a single patient example.** This figure presents AF burden values as bars and AF burden moving averages as lines for each follow-up day between the day after device implantation and the day before cardiovascular hospitalization. First AF detection and moving average cross-over events trigger different windows of risk. Trend A represents a window of sinus rhythm before the patient’s first device-detected AF event (baseline risk). Trend B is triggered when the 21-day moving average of AF burden crosses below its historical average (below average risk). Trend C is triggered when the 21-day moving average of AF burden crosses above its historical average (above average risk). Moving averages are adjusted downward by 5 minutes for display purposes. AT indicates atrial tachycardia; CMA, cumulative moving average; and SMA, simple moving average.

Our random forest methodology evaluated dynamic trends across 5 diagnostic parameters for the near-term risk of CVH. The most likely trends (ie, the decision tree rules that occurred most frequently across the continuum of risk) were defined by AF burden (Supplemental Methods S2). Table 2 presents a definition for each AF burden trend. Trend A is defined by an average AF burden from implant to follow-up of <1%. With little history of AF burden at follow-up, the trend describes a window of sinus rhythm before the first onset of AT/AF (78% of occurrences) and the window of prolonged sinus rhythm after an episode of device-detected AT/AF (22% of occurrences). Trend B is defined by a history of AF burden where the 21-day average is trending below the average burden calculated from implant. Trend B describes a window of lower burden amount following a period of elevated burden. Conversely, Trend C is defined by a history of AF burden where the 21-day average is trending at or above the average burden calculated from implant. This trend describes a relative spike or above-average trend in daily AF burden. Figure 2 presents an example of these trends mapped to a single patient.

**Table 2.**
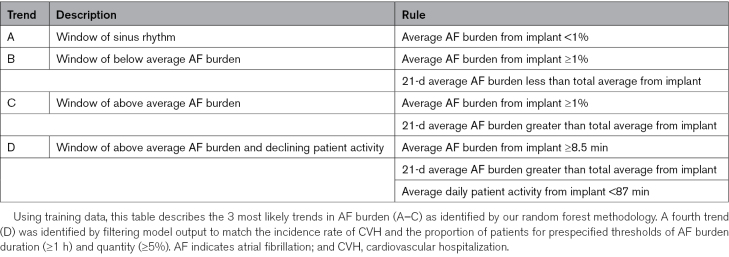
AF Burden Trends

A match to prespecified thresholds by incidence rate of CVH and percent of patients defined a fourth trend. The proportion of patients and the corresponding incidence rate of CVH were calculated on the training data for prespecified AF burden duration (≥1 hour) and quantity (≥5%) thresholds. Log RR was 0.386 and 0.369, and patient percent was 7.6% and 12.9% for the respective thresholds. Table 2, Trend D presents output where the log RR was >0.369 and the percent of patients was >12.9% (our match to prespecified thresholds). Derived from machine learning, trend D is a subset of trend C that is further defined by a historical average AF burden >8.5 minutes and a patient’s average daily activity from implant below 87 minutes. This trend describes a relative spike or above-average trend in daily AF burden for patients with an average of 8.5 minutes of daily burden and decreasing activity levels. When applied to the training set, a trend of increasing burden and decreasing patient activity (trend D) was selected 8.3% of the time and had a log RR of 0.369, a value that was not statistically different from duration or quantity risk ratios (Poisson regression, *P*>0.5 for all coefficients).

### Risk Stratification

**Figure 3.**
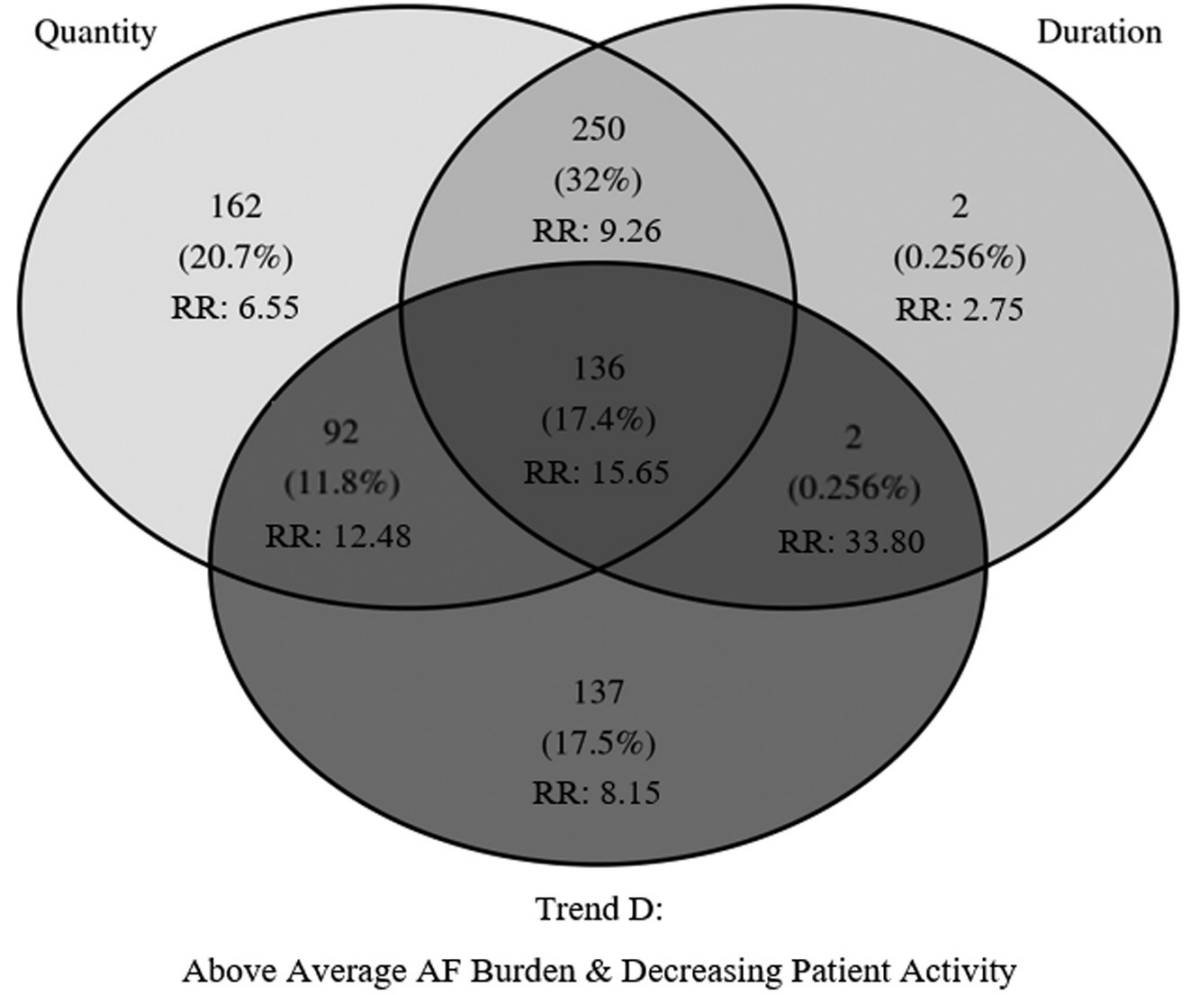
**Venn diagram of cardiovascular hospitalization (CVH) events by atrial fibrillation (AF) burden criteria.** This Venn diagram shows the number of CVH events (%) and relative risk for the intersection of AF burden duration (≥1 hour), quantity (≥5%), and Trend D (increasing burden with decreasing patient activity) sets. Trend D captures 137 mutually exclusive CVH events, yielding a 21% (137/644) increase in detection over clinical criteria alone. The size of the Venn diagram circles and their overlay are kept constant for ease of presentation and thus do not represent the relative contribution of each set. RR indicates relative risk.

Table 3 presents validation statistics for AF burden duration, quantity, and trend thresholds, including their intersecting sets. AF burden trends (A–C) provided clear segmentation of event risk (B versus A, RR, 4.49 [95% CI, 3.74–5.40]; C versus A, RR, 8.41 [95% CI, 7.00–10.11]). Including a daily patient activity threshold (D) provided more specific coverage with the greatest risk for CVH among AF burden trends (D versus A, RR, 11.15 [95% CI, 9.10–13.65]). Approximately 51% (5114/10 017) of increasing burden and decreasing patient activity (trend D) thresholds were mutually exclusive to duration and quantity thresholds and represented a 21% (137/644) increase in event yield (Figure 3). About 20% of patients experienced the trend D threshold at an expected rate of 15.9% of follow-up days, or 58 days per year. For criteria with at least 100 threshold events, the intersection of increasing burden and decreasing patient activity, duration, and quantity thresholds was associated with the greatest risk for CVH (RR, 15.65 [95% CI, 12.31–19.91]). This result was significantly different from duration and quantity thresholds alone (RR, 9.26 [95% CI, 7.47–11.48]; *P*<0.001). AUROC improved from 0.55 (95% CI, 0.55–0.56) for duration and quantity criteria to 0.66 ([95% CI, 0.65–0.67]; *P*<0.001) when increasing burden and decreasing patient activity was added as a third criterion, representing an 11% absolute and 20% relative increase in predictive power.

**Table 3.**
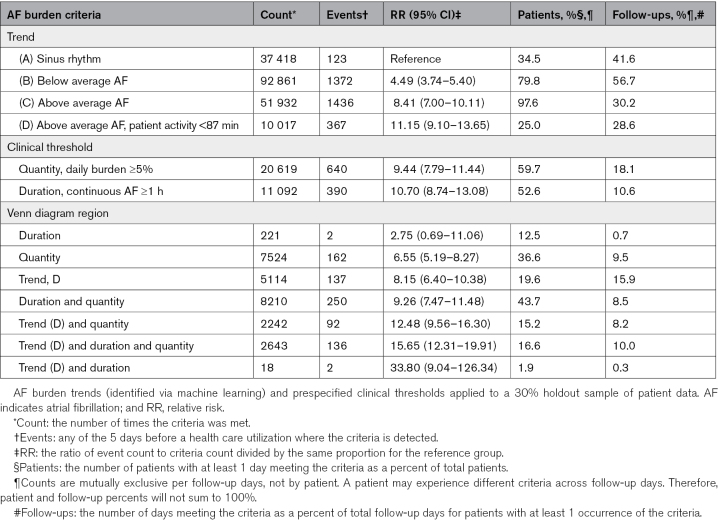
Validation Statistics for Different AF Burden Criteria

### Time in AT/AF

**Figure 4.**
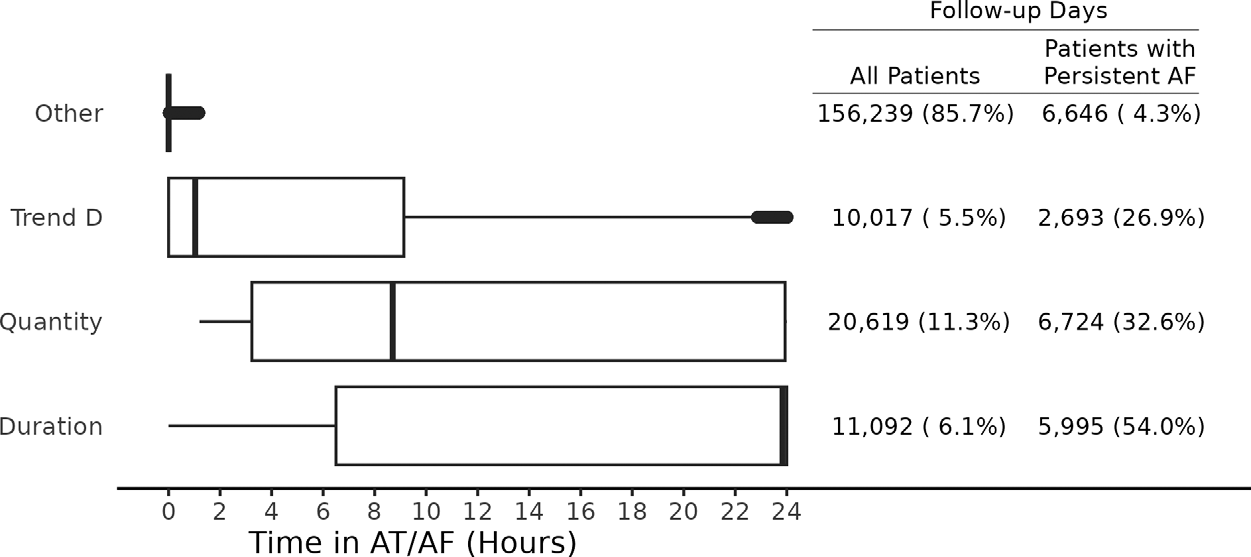
**Distribution of daily atrial tachycardia (AT)/atrial fibrillation (AF) amount by AF burden criteria.** Using validation data, this plot visualizes the differences in daily AT/AF amount by AF burden threshold. Follow-up days are counted when the criteria or trend is met for the respective AF burden threshold and presented as a percent of total follow-ups. Of these days, follow-up days are further counted only for patients with persistent AF.

The distribution of time in AT/AF differed by AF burden criteria for a validation sample of 182 211 follow-up days. The median (25th, 75th percentile) time in AT/AF for an increasing burden and decreasing patient activity trend was 1.0 (0–9.1) versus 8.7 (3.2–24.0) hours for quantity and 23.8 hours (6.5–24.0) for duration. Distributions of these data are shown in Figure 4 along with the percent of follow-up days for patients in persistent AF. Of the 3 AF burden thresholds, increasing burden and decreasing patient activity had the lowest rate of follow-up days in persistent AF (26.9% versus duration 54.0%, and quantity 32.6%; *P*<0.001) and the greatest incidence rate of CVH (3.7% versus duration 3.5% and quantity 3.1%; *P*=0.020).

## Discussion

In the present study, we evaluated trends associated with near-term CVH for patients with an ICM by linking device-detected diagnostic measures with administrative claims data. Within different time windows, the amount and change in daily AT/AF burden, patient activity, ventricular rate, and heart rate variability were used to predict the near-term risk of CVH. Our approach involves a change-from-baseline method, which personalizes the RR of CVH against changes in ICM-specific diagnostics for each patient. This allows for time-updated risk calculations at each daily follow-up through continuous monitoring. This distinction is important because, at a population level, we can observe that an overall increase in AF burden is associated with an elevated risk of CVH. However, this overlooks the dynamic changes in AF burden alongside other diagnostic parameters at the individual level. We demonstrated that this dynamic risk stratification approach enables us to monitor patients over time and provide real-time risk assessments within a shorter time frame.

Although we defined, a priori, a set of windows for tracking changes in ICM diagnostic parameters, we allowed machine learning to identify the dynamic changes or trends associated with the risk of CVH. Our results showed that trends in AF burden were the most frequently selected for risk detection among all diagnostic parameters and that the 21-day moving average was the optimal time window for risk stratification, regardless of the indication for ICM implantation (AF management, suspected AF, or cryptogenic stroke). Using a 21-day moving average, within-patient comparisons demonstrated that individuals with above-average AF burden and decreasing levels of activity stratified near-term risk of CVH as well as prespecified AF burden duration (≥1 hour) and quantity (≥5%) thresholds.

To summarize, there are 3 major findings from this work. First, machine learning was able to describe dynamic changes in AF burden associated with the risk of CVH. Second, the amount of change in AF burden needed to signal risk may depend on a patient’s baseline history of AF burden. Last, dynamic changes in AF burden add predictive power to conventional risk stratification thresholds of AF burden duration and quantity. Our findings have important implications for future research attempting to define and use AF burden risk thresholds for the management of AF-related outcomes.

Though there is strong evidence that patients in persistent AF are at a higher risk of stroke than those with paroxysmal AF,^4,8–10^ and emerging evidence that the same is true for CVH,^3^ there is significant heterogeneity in the definition of AF burden used in these studies. Accordingly, it remains unclear whether a single prolonged episode has more prognostic value than the total quantity of AF burden.^1^ Indeed, 2 patients with the same AF burden amount but different temporal densities of AF burden will have different probabilities for AF detection using intermittent monitoring.^11^ Thus, there is an opportunity to construct personalized risk thresholds from continuous measures of AF burden via long-term cardiac monitoring devices.

In this analysis, machine learning on data from ICMs and linked outcomes demonstrates that dynamic changes in AF burden are associated with the risk of CVH. While a recent study has shown that changes in AF burden add prognostic value for the risk stratification of stroke,^12^ this is the first study to identify and describe dynamic changes in AF burden, using machine learning, that may improve the near-term prediction of CVH. This finding confirms the potential for a more individualized approach to AF management by redefining risk thresholds from the population to the patient.

At a patient level, there is a unique dynamic between the 21-day moving average of AF burden and its change over the average daily burden (baseline). The 21-day window, identified by machine learning analysis, coincides with what is known about the temporal proximity of AF and stroke.^13,14^ It is also short enough for tracking meaningful changes in AF burden over time and for creating thresholds of below- and above-average burden when compared with a patient’s daily average. The continuum of average patient burden and its risk stratification of CVH suggests that the general association between increasing burden and increasing risk, historically defined at the population level,^3,15–19^ is also consistent at the individual patient level. The increase in AF burden that portends increased risk in the future depends upon the baseline AF burden in that individual. In application, this type of change-from-baseline assessment presents the possibility of personalized risk prediction for AF-related care.

While there is no universal consensus on the baseline amount of AF burden needed to signal risk for CVH, machine learning analysis shows a minimum average daily burden amount of 8.5 minutes. Practically, this means a patient with a single 255-minute episode within the first 30 days after device insertion has the same risk of CVH as a patient who averages 8.5 minutes every day for that same period. The implication is that both a single episode of AF and the total quantity of AF have prognostic value provided they meet some daily average threshold. Tracking average daily burden via continuous monitoring is a simple way to resolve this uncertainty and to provide additional prognostic value for the risk management of CVH.

Interestingly, tracking a change in AF burden over short time periods for deviations from a patient’s historical baseline adds prognostic value to prespecified thresholds of duration or quantity. In the present study, tracking daily changes in the 21-day average of AF burden for patients with declining activity and <1 hour of daily AT/AF identified 21% more CVH events over the detection of duration and quantity thresholds, events that would have been missed when using criteria requiring >1 hour of AF. And while a reported AUROC of 0.66 is relatively low for remote predictive modeling,^20^ it represents an 11% absolute and 20% relative increase over matched comparators. Without matching, internal results (not shown) present a more complex trend involving AF burden, patient activity, and heart rate variability, with a mean AUROC of 0.75. Adding dynamic trends from the ECG waveform results in a mean AUROC of 0.80. In general, tracking dynamic trends in ICM-detected diagnostic patterns provides a method for adding prognostic value to prespecified thresholds of duration or quantity.

Our approach also added specificity to absolute thresholds: data showed a 45% to 65% increase in RR of CVH for patients meeting duration, quantity, and AF burden trend thresholds at the same time. Brief asymptomatic episodes of atrial arrhythmias are common and have less certain clinical significance for patient outcomes, causing a lack of standardization among definitions and metrics used for diagnosis and treatment.^21^ A change-from-baseline approach using dynamic trends in AF burden overcomes this limitation as an independent predictor for near-term CVH risk. It also modifies the current AF burden paradigm—that increasing AF burden is associated with increasing cardiovascular event risk—with a novel calculus for measuring changes in AF burden over time, in turn allowing for a more precise standardization of definitions across the spectrum of subclinical AF.

Our findings offer 3 considerations for how AF burden may better inform patient care. First, given the lack of clearly defined thresholds of AF burden at which point intervention should be implemented, the use of historical and 21-day average burden measures in place of population-level metrics may provide needed decision-making support for personalized patient care. Highlighting when the 21-day average is above the historical average is a simple modification that would give clinicians an objective measure for patient-specific risk assessments. Second, in the absence of a definitive AF burden threshold, it has been shown that the duration and quantity of AF burden can improve the risk stratification for stroke when combined with CHA_2_DS_2_-VASc scores.^22,23^ Given its complementary nature, a change in 21-day average burden above its historical average could be used to confirm elevated risk of stroke for patients with < 6 minutes of daily AF duration and a CHA_2_DS_2_-VASc score >2. Alternatively, the crossing of a 21-day average burden over its historical baseline could inform the timing of therapies such as lifestyle modification or rate/rhythm control therapies. The large variation in clinical actions among care providers, for instance with treatment discrepancies among individuals receiving oral anticoagulation after device-detected AF,^24^ acts as a foundation for equally variable results. Accordingly, we believe these considerations of how to use a change-from-baseline approach for the management of AF-related outcomes may better help guide clinical judgement, although a prospective study to test the efficacy of this management style should be undertaken.

### Limitations

There are several limitations to the current study. First, all patients in the analysis had an ICM implanted for the clinical care of AF management, suspected AF, or evaluation of cryptogenic stroke. Thus, ICM-specific trends may not generalize to patients with other device indications, other cardiac implanted electronic devices, or other diagnostic devices like wearables. Second, arrhythmia events were classified by the device algorithm and not adjudicated by clinical experts. While AF burden was not corrected for inappropriate AF episode detection, it has been observed in earlier studies that inappropriate detections are primarily short-duration episodes and do not affect burden significantly.^25^ Third, this study utilized administrative claims for outcomes assessment, including both planned and unplanned CVHs. This is a broad definition that does not distinguish between hospitalizations related to AF and those not related to AF. Accordingly, increasing trends in AF burden should be regarded as a predictive marker for CVH rather than a causal mechanism for the purposes of this analysis. Fourth, the patient cohort included a mix of ICM device indications and treatments. While this increases the generalizability of the results to a real-world setting, it should be kept in mind that the results may not generalize optimally to patients undergoing a specific therapy or intervention. Finally, the AF burden trend modeling had 2 limitations. One was only including patients who had at least 1 day of device-detected AF burden. This look-forward inclusion criterion means our reference category for RR (trend A) is only valid once the device documents AF for the first time. It is likely that some ICM patients may never have any device-detected burden from the implant and, thus, have a different near-term risk for CVH than what our reference category suggests. While this may not present as a limitation for the clinical management of patients with a known history of AF, the baseline RR of CVH for patients without such a history could be different. The second limitation to the modeling approach was how the validation sample was defined. The 30% of patients used to validate CVH risk prediction (Table 3) were drawn from the same patients used in the random forest modeling. This is not standard procedure when evaluating a predictive model. However, our objectives were to define an AF burden trend that could predict CVH risk and to compare its performance to prespecified measures of duration and quantity. We accomplished this by defining trend D (increasing burden and decreasing patient activity) on a 70% random sample of random forest patients and then evaluating its performance on the remaining 30% using comparative measures of RR and AUROC. Thus, while bias was mitigated using random partitioning or held constant across comparisons, validated CVH event rates may be overstated and should not be regarded as absolute.

### Conclusions

In this study of patients with documented AF, we identified dynamic AF burden trends that could predict the risk of near-term CVH, thereby offering additional yield in CVH detection and an increase in RR over existing thresholds of duration and quantity. Our results and implications for future research support current guidance that continuous monitoring technologies might allow for the detection of more precise changes in AF burden over time. The prospective DEFINE AFIB study has enrolled hundreds of patients with a history of AF monitored by ICM to evaluate whether trends in ICM device-collected AF burden can appropriately stratify patients for adverse clinical outcomes, including stroke and CVH (REGISTRATION: URL: https://www.clinicaltrials.gov; Unique identifier: NCT04926857).

## ARTICLE INFORMATION

### Sources of Funding

This analysis was funded by Medtronic.

### Disclosures

Dr Peacock serves as a consultant to Medtronic and Biotronik. E.J. Stanelle, Dr Johnson, and R. Kanwar are employed by Medtronic. D. Soderlund was formerly employed by Medtronic. Dr Hylek serves as a consultant for Bayer, Bristol Myers Squibb, Ionis, Janssen, and Pfizer, receives research grants from Abbott, Anthos Therapeutics, and Medtronic, and receives honoraria from Boehringer Ingelheim. Dr Lakkireddy serves as a consultant to Medtronic, Boston Scientific, Abbott, Atricure, AltaThera, Acutus, and AliveCor. Dr Mittal serves as a consultant to Abbott, Boston Scientific, and Medtronic. Dr Passman is supported by UG3HL165065 from NHLBI 18SFRN34250013 from the American Heart Association (AHA), receives a grant for clinical research from Abbott, serves as a consultant to Medtronic, Janssen Pharmaceuticals, and Abbott, and receives royalties from UpToDate. Dr Russo serves as a consultant for Abbott, Atricure, Bayer, Biosense Webster, Boston Scientific, Medtronic, and PaceMate receives grants for clinical research from Boston Scientific, Kestra, Medilynx, and Medtronic and receives honoraria from Biotronik, Bristol Myers Squibb, Pfizer, Medtronic, and Sanofi. Dr Piccini is supported by R01AG074185 from the National Institute on Aging receives grants for clinical research from Abbott, AHA, the Association for the Advancement of Medical Instrumentation, Bayer, Boston Scientific, iRhythm, and Philips serves as a consultant to Abbott, AbbVie, Ablacon, AltaThera, ARCA Biopharma, Biotronik, Boston Scientific, Bristol Myers Squibb, LivaNova, Medtronic, Milestone, Electrophysiology Frontiers, Pfizer, Sanofi, Philips, and UptoDate. The other author report no conflicts.

### Supplemental Material

Supplemental Methods S1 and S2

Tables S1–S3

Figure S1

References 26–31

## Supplementary Material


