# Supplementary material for: Using Atrial Fibrillation Burden Trends and Machine Learning to Predict Near-Term Risk of Cardiovascular Hospitalization
Source: Circ Arrhythm Electrophysiol. 2024 Oct 24;17(11):e012991. doi: 10.1161/CIRCEP.124.012991 (PMC11575902; doi:10.1161/CIRCEP.124.012991)

Supplemental Material

Supplemental Methods 1: Random forest methodology

A case-control design was used at the level of follow-up period, allowing for case-crossover for patients with CVH. Our dependent variable, a follow-up period with CVH, was compared with all other follow-up periods for patient cases and controls, resulting in a rare event rate of 0.33%. To balance the data, CVH events were oversampled by labeling the five days prior to an occurrence. Control follow-ups were then randomly undersampled to match the number of oversampled follow-up cases. For patients who experienced CVH, follow-up ended on the day prior to the occurrence to prevent the use of device measurements taken on the same day but after CVH happened, a situation that would introduce *look ahead* bias into the modeling. Thus, our goal was to accurately predict CVH five days in advance using all labeled follow-up cases and a random sample of follow-up controls.

A recursive partitioning & regression tree algorithm (RPART)[26, 27] was used to predict which follow-up days had an occurrence of CVH using device parameter values and dynamic trends as predictors. Although over/undersampling methods are well documented for managing imbalanced data,[28-30] including the prediction for incident AF,[31] we recognized the oversampling bias in our design and used a bootstrapping routine with 3,000 repetitions to remove the bias and improve classifier accuracy. For each bootstrap iteration:

1. Patients were randomly partitioned into training (70%) and validation (30%) sets.

2. Follow-up controls were randomly sampled without replacement to equal the number of follow-up cases, resulting in a balanced training set.
3. An exhaustive classification tree with a minimum terminal node size equal to 80, a maximum depth equal to 8, and a complexity parameter equal to 0 was fit to the balanced training set.
4. The misclassification rate for each subtree in Step 3 was calculated using 10-fold cross validation. The exhaustive tree was then pruned back to the subtree with the lowest misclassification rate.
5. Split information from the model fit in Step 4 were saved.
6. The unbalanced validation set was classified using the model fit from Step 4.
7. Classification statistics for each rule from Step 6 were saved.

Random forest methodology diagram.

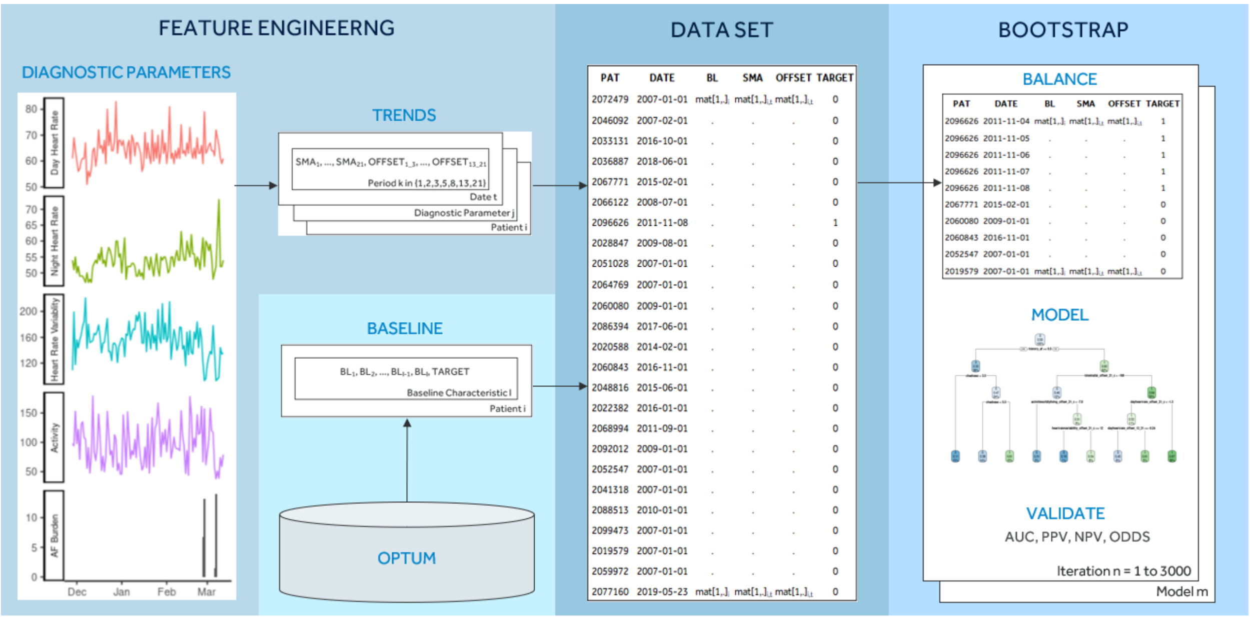

Supplemental Methods 2: AF burden trend modeling

Split information for each bootstrapped classification tree was saved as a set of rules. Each rule was defined by a predictor name, a comparison (< or ≥), and an index value. For example, a tree with only one split at an AF burden amount of five hours would have two rules: [AF burden, <, 5] and [AF burden, ≥, 5]. Patient count and incidence rate of CVH were calculated for each rule. Each rule was saved as a separate entry if a terminal node had multiple rules. In such a case, the incidence rate and patient count would be the same for all rules belonging to the same terminal node. Rules from all bootstraps were combined and a scatterplot showing the relationship between CVH incidence rate and the percent of patients was used to identify frequently occurring rules or *trends* in AF burden that stratified CVH risk during model development. The algorithm for defining these trends was:

1. Visually identify areas in the scatterplot with a local maximum in patient percent.
2. If the area is unique to Time in AT/AF, define rectangular coordinates for event risk and patient percent that enclose the area.
3. If the area is not unique to Time in AT/AF, then
    i. Set the upper and lower boundary for patient percent equal to the local maximum
    ii. Subtract 0.01 from the lower boundary for patient percent.
    iii. Set the lower and upper boundaries for incidence rate to the respective locations where the scatterplot intersects with the lower patient percent boundary defined in the preceding step.

4. Select nodes within the rectangular area.
5. Group by the pair [predictor name, comparison].
6. Calculate the number of times each pair is selected, the number of times each pair is selected as a percent of all bootstrapped classification trees, and the mean of [index values].
7. If the area is not unique to Time in AT/AF, repeat Steps 3.ii - 6 until the modal predictor is selected in at least 10% of all classification trees.
8. Rank order rules [predictor, comparison, mean index value] in descending order by selection rate.
9. Identify the *elbow* in selection rate (i.e. where the selection rate drops by approximately 50%) for rules that occur at least 25 times.
10. Define an AF burden trend as the rules having a selection rate above the elbow point identified in the preceding step.

AF burden trend modeling diagram.

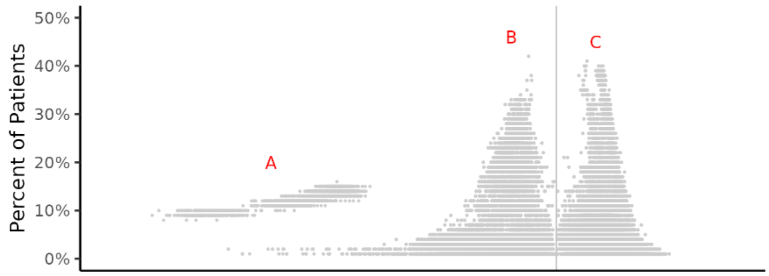

Step 1: Visualize rules by CVH incidence rate and patient percent. Identify modal areas where rules occur most frequently.

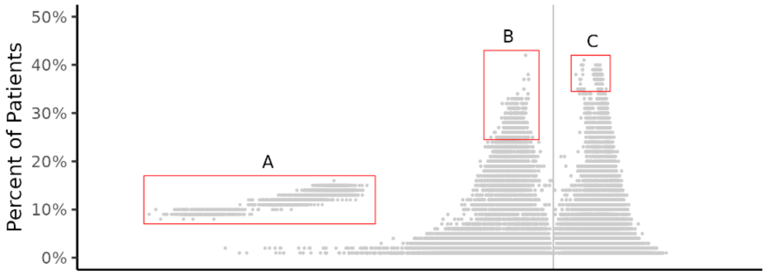

Step 2: Select coordinates around modal area and summarize the frequency of each predictor. Expand coordinates until a single predictor is selected at least 10% of the time.

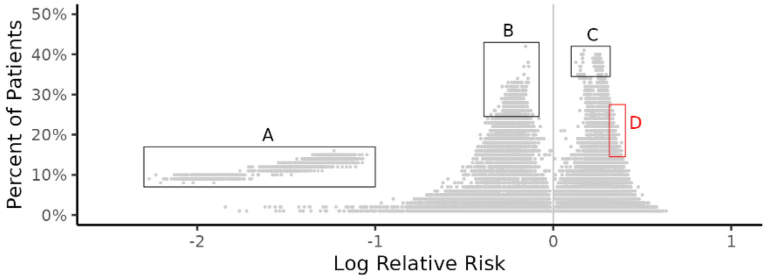

Step 3: Identify an area that has the same risk and patient coverage as clinical thresholds.

Area	Predictor	Comparison	Mean	Count	Selection
**A**	**timeinafat_c**	**<**	**857**	**3,000**	**100.00%**
	timeinafat_offset_3_c	>=	-173	19	0.63%
	timeinafat_offset_5_c	>=	-154	10	0.33%
	timeinafat_offset_2_c	>=	-196	2	0.07%
**B**	**timeinafat_c**	**>=**	**805**	**712**	**23.73%**
	**timeinafat_offset_21_c**	**<**	**-647**	**581**	**19.37%**
	**timeinafat_offset_1_21**	**>=**	**-84,028**	**570**	**19.00%**
	timeinafat	<	1,276,505	309	10.30%
	timeinafat_c	<	3,777,456	183	6.10%
**C**	**timeinafat_c**	**>=**	**917**	**1,191**	**39.70%**
	**timeinafat_offset_21_c**	**>=**	**-649**	**1,134**	**37.80%**
	activitiesofdlyliving_offset_21_c	>=	-12	197	6.57%
	heartratevariability_offset_21_c	>=	-7	68	2.27%
	timeinafat	<	882,857	56	1.87%
**D**	**timeinafat_c**	**>=**	**510,453**	**59**	**1.97%**
	**timeinafat_offset_21_c**	**>=**	**-28,974**	**37**	**1.23%**
	**activitiesofdlyliving_c**	**<**	**87**	**25**	**0.83%**
	timeinafat	>=	648,000	20	0.67%
	heartratevariability_offset_21_c	>=	-6	18	0.60%

Step 4: For each area, we identify the predictor where the selection rate drops by approximately 50% or more. The predictors above this change point define the AF burden trend for each area.

Supplemental Table S1: Diagnosis and procedure codes

Disease	Type	ICD	Codes
Ablation	proc	09	3734, 93650, 93651, 93652, 93653, 93654, 93655, 93656, 93657
		10	02583ZZ
Atrial Fibrillation	diag	09	427.31
		10	I48.0, I48.1, I48.2, I48.91
Cardiovascular Hospitalization	diag	09	390.*, 391.*, 392.*, 393.*, 394.*, 395.*, 396.*, 397.*, 398.*, 401.*, 402.*, 403.*, 404.*, 405.*, 410.*, 411.*, 412.*, 413.*, 414.*, 415.*, 416.*, 417.*, 420.*, 421.*, 422.*, 423.*, 424.*, 425.*, 426.*, 427.*, 428.*, 429.*, 430.*, 431.*, 432.*, 433.*, 434.*, 435.*, 436.*, 437.*, 438.*, 440.*, 441.*, 442.*, 443.*, 444.*, 445.*, 446.*, 447.*, 448.*, 449.*, 451.*, 452.*, 453.*, 454.*, 455.*, 456.*, 457.*, 458.*, 459.*
		10	I00.*, I01.*, I02.*, I05.*, I06.*, I07.*, I08.*, I09.*, I10.*, I11.*, I12.*, I13.*, I14.*, I15.*, I16.*, I20.*, I21.*, I22.*, I23.*, I24.*, I25.*, I30.*, I31.*, I32.*, I33.*, I34.*, I35.*, I36.*, I37.*, I38.*, I39.*, I40.*, I41.*, I42.*, I43.*, I44.*, I45.*, I46.*, I47.*, I48.*, I49.*, I50.*, I51.*, I52.*, I60.*, I61.*, I62.*, I63.*, I64.*, I65.*, I66.*, I67.*, I68.*, I69.*, I70.*, I71.*, I72.*, I73.*, I74.*, I75.*, I76.*, I77.*, I78.*, I79.*, I80.*, I81.*, I82.*, I83.*, I84.*, I85.*, I86.*, I87.*, I88.*, I89.*, I95.*, I96.*, I97.*, I98.*, I99.*
Chronic Kidney Disease	diag	09	585*
		10	N18*
COPD	diag	09	491.21

Disease	Type	ICD	Codes
		10	J44.1
Coronary Artery Disease	diag	09	414.00, 414.01
		10	I25.10
Diabetes Mellitus	diag	09	249*, 250*, 3572, 36201, 36202, 36203, 36204, 36205, 36206, 36641
		10	E08*, E09*, E10*, E11*, E13*
Heart Failure	diag	09	39891, 40201, 40211, 40291, 40401, 40403, 40411, 40413, 40491, 40493, 4280, 4281, 42820, 42821, 42822, 42823, 42830, 42831, 42832, 42833, 42840, 42841, 42842, 42843, 4289
		10	I0981, I110, I130, I132, I501, I5020, I5021, I5022, I5023, I5030, I5031, I5032, I5033, I5040, I5041, I5042, I5043, I50810, I50811, I50812, I50813, I50814, I5082, I5083, I5084, I5089, I509
Hypertension	diag	09	36211, 4010, 4011, 4019, 40200, 40201, 40210, 40211, 40290, 40291, 40300, 40301, 40310, 40311, 40390, 40391, 40400, 40401, 40402, 40403, 40410, 40411, 40412, 40413, 40490, 40491, 40492, 40493, 40501, 40509, 40511, 40519, 40591, 40599, 4372
		10	H35031, H35032, H35033, H35039, I10, I110, I119, I120, I129, I130, I1310, I1311, I132, I150, I151, I152, I158, I159, I674, N262
Hyperthyroidism	diag	09	242.90
		10	E05.90
Hypothyroidism	diag	09	244.9

Disease	Type	ICD	Codes
		10	E03.9
Ischemic Stroke	diag	09	433.01, 433.11, 433.21, 433.31, 433.81, 433.91, 434.01, 434.11, 434.91, 436*, 997.02
		10	I63.6, I63.8*, I63.9, I6300, I63011, I63012, I63013, I63019, I6302, I63031, I63032, I63033, I63039, I6309, I6310, I63111, I63112, I63113, I63119, I6312, I63131, I63132, I63133, I63139, I6319, I6320, I63211, I63212, I63213, I63219, I6322, I63231, I63232, I63233, I63239, I6329, I6330, I63311, I63312, I63313, I63319, I63321, I63322, I63323, I63329, I63331, I63332, I63333, I63339, I63341, I63342, I63343, I63349, I6339, I6340, I63411, I63412, I63413, I63419, I63421, I63422, I63423, I63429, I63431, I63432, I63433, I63439, I63441, I63442, I63443, I63449, I6349, I6350, I63511, I63512, I63513, I63519, I63521, I63522, I63523, I63529, I63531, I63532, I63533, I63539, I63541, I63542, I63543, I63549, I6359, I64, I97.810, I97.811, I97.820, I97.821
Myocardial Infarction	diag	09	37.66, 410, 4100, 41000, 41001, 41002, 4101, 41010, 41011, 41012, 4102, 41020, 41021, 41022, 4103, 41030, 41031, 41032, 4104, 41040, 41041, 41042, 4105, 41050, 41051, 41052, 4106, 41060, 41061, 41062, 4107, 41070, 41071, 41072, 4108, 41080, 41081, 41082, 4109, 41090, 41091, 41092, 412, V43.21
		10	I21, I21.0, I21.01, I21.02, I21.09, I21.1, I21.11, I21.19, I21.2, I21.21, I21.29, I21.3, I21.4, I22, I22.0, I22.1, I22.2, I22.8, I22.9, I23, I23.0, I23.1, I23.2, I23.3, I23.4, I23.5, I23.6, Z95.811
Sleep Apnea	diag	09	32720, 32721, 32723, 32729
		10	G47.30, G47.31, G47.33, G47.39

Disease	Type	ICD	Codes
Stroke/TIA	diag	09	430, 431, 43301, 43311, 43321, 43331, 43381, 43391, 43400, 43401, 43410, 43411, 43490, 43491, 4350, 4351, 4353, 4358, 4359, 436, 800*, 801*, 802*, 803*, 804*, 850*, 851*, 852*, 853*, 8541, 99702, V57*
		10	G450, G451, G452, G458, G459, G460, G461, G462, G463, G464, G465, G466, G467, G468, G9731, G9732, I6000, I6001, I6002, I6010, I6011, I6012, I6020, I6021, I6022, I6030, I6031, I6032, I604, I6050, I6051, I6052, I606, I607, I608, I609, I610, I611, I612, I613, I614, I615, I616, I618, I619, I6300, I63011, I63012, I63013, I63019, I6302, I63031, I63032, I63039, I6309, I6310, I63111, I63112, I63119, I6312, I63131, I63132, I63139, I6319, I6320, I63211, I63212, I63213, I63219, I6322, I63231, I63232, I63233, I63239, I6329, I6330, I63311, I63312, I63313, I63319, I63321, I63322, I63323, I63329, I63331, I63332, I63333, I63339, I63341, I63342, I63343, I63349, I6339, I6340, I63411, I63412, I63413, I63419, I63421, I63422, I63423, I63429, I63431, I63432, I63433, I63439, I63441, I63442, I63443, I63449, I6349, I6350, I63511, I63512, I63513, I63519, I63521, I63522, I63523, I63529, I63531, I63532, I63533, I63539, I63541, I63542, I63543, I63549, I6359, I636, I638, I639, I6601, I6602, I6603, I6609, I6611, I6612, I6613, I6619, I6621, I6622, I6623, I6629, I663, I668, I669, I67841, I67848, I6789, I97810, I97811, I97820, I97821, S0190XA, S02*, S06*
Systemic Embolism	diag	09	444.01, 444.09, 444.1, 444.21, 444.22, 444.81, 444.89, 444.9
		10	I74.01, I74.09, I74.10, I74.11, I74.19, I74.2, I74.3, I74.4, I74.5, I74.8, I74.9
Valvular Heart Disease	diag	09	394*, 395*, 396*, 397*

Disease	Type	ICD	Codes
		10	I34*, I35*, I37*
Vascular Disease	diag	09	4400, 4401, 4402, 44020, 44021, 44022, 44023, 44029, 4404, 4438, 44381, 44382, 44389, 4439
		10	I700, E0851, E0852, E0951, E0952, E1051, E1052, E1151, E1152, E1351, E1352, I701, I70201, I70202, I70203, I70208, I70209, I70211, I70212, I70213, I70218, I70219, I70221, I70222, I70223, I70228, I70229, I70231, I70232, I70233, I70234, I70235, I70238, I70239, I70241, I70242, I70243, I70244, I70245, I70248, I70249, I7025, I70291, I70292, I70293, I70298, I70299, I7092, I7381, I7389, I739, I791, I798

ICD indicates International Classification of Diseases.

Supplemental Table S2: Oral Anticoagulants

Drug Name
ACENOCOUMAROL, APIXABAN, BETRIXABAN, COUMADIN, DABIGATRAN, EDOXABAN, ELIQUIS, FLUINDIONE, LIXIANA, PHENPROCOUMON, PRADAXA, RIVAROXABAN, SAVAYSA, WARFARIN, XARELTO

Supplemental Table S3: Diagnostic Related Group

Cardiovascular Hospitalization
215, 216, 217, 218, 219, 220, 221, 222, 223, 224, 225, 226, 227, 228, 229, 230, 231, 232, 233, 234, 235, 236, 237, 238, 239, 240, 241, 242, 243, 244, 245, 246, 247, 248, 249, 250, 251, 252, 253, 254, 255, 256, 257, 258, 259, 260, 261, 262, 263, 264, 265, 266, 267, 268, 269, 270, 271, 272, 273, 274, 275, 276, 277, 278, 279, 280, 281, 282, 283, 284, 285, 286, 287, 288, 289, 290, 291, 292, 293, 294, 295, 296, 297, 298, 299, 300, 301, 302, 303, 304, 305, 306, 307, 308, 309, 310, 311, 312, 313, 314, 315, 316

Supplemental Figure S1: Cohort select diagram

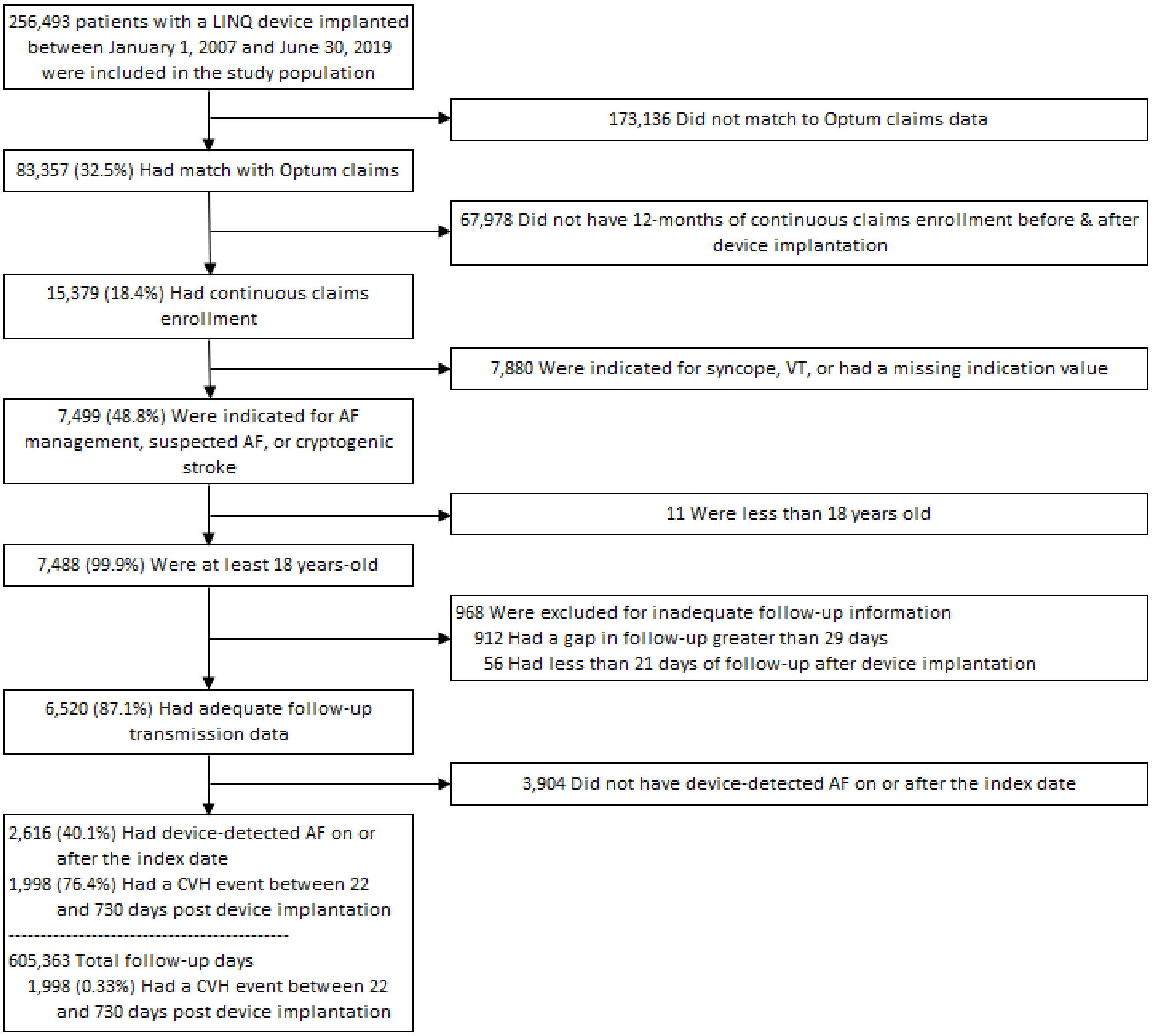

Supplement: Supplementary file 1 [file hae-17-e012991-s001.pdf]
